# Biological Evaluation of *Pupalia lappacea* for Antidiabetic, Antiadipogenic, and Hypolipidemic Activity Both *In Vitro* and *In Vivo*

**DOI:** 10.1155/2016/1062430

**Published:** 2016-01-28

**Authors:** Vivek Kumar, Parag Jain, Kalpana Rathore, Zabeer Ahmed

**Affiliations:** ^1^Department of Pharmacology, School of Pharmacy, Babu Banarasi Das University, Lucknow, Uttar Pradesh 226005, India; ^2^Department of Pharmacology, SLT Institute of Pharmaceutical Sciences, Guru Ghasidas Vishwavidyalaya, Bilaspur, Chhattisgarh 495334, India; ^3^University Teaching Department, Sarguja University, Ambikapur, Chhattisgarh 497001, India; ^4^Department of Pharmacology, Indian Institute of Integrative Medicine, Jammu, Jammu and Kashmir 180001, India

## Abstract

*Objective*. The present study assesses the effect of* Pupalia lappacea* (L.) Juss. (Amaranthaceae) (*PL*) leaves ethanolic extract on adipocytes, blood glucose level, and lipid level in streptozotocin (STZ) induced diabetic rats.* Materials and Methods*. Male Albino rats were rendered diabetic by a single moderately sized dose of STZ (45 mg/kg, intraperitoneally) at once before starting the treatment. Animals were divided into five groups: normoglycemic control, diabetic control, reference group (glibenclamide, 5.0 mg/kg), AS001 (250 mg/kg extract), and AS002 (500 mg/kg extract) each containing six animals for* in vivo* study. Antidiabetic and hypolipidemic activity of extract were determined by* in vivo* method on STZ induced diabetic rats. Antiadipogenic activity was determined by* in vitro* method on 3T3-L1 cell line in comparison to simvastatin as reference drug.* Result*. The extract showed significant fall in fasting serum glucose (FSG), that is, 234.68 and 211.61 mg/dL, in STZ induced diabetic animals for dose groups AS001 and AS002, respectively. The* PL* extract also exhibited noteworthy antiadipogenic activity on 3T3-L1 cell line. The value of inhibitory concentration (IC50) of* PL* extract to reduce adipocyte cells was found to be 662.14 *μ*g/mL.* Conclusion*. The* PL* extract exhibited significant antiadipogenic, antidiabetic, and hypolipidemic activities.

## 1. Introduction

The plant* Pupalia lappacea* (L) Juss. (Amaranthaceae)* (PL)*, commonly known as Forest Burr or Creeping cock's comb [1], is a perennial herb, erect or prostrate and sprawling, 60–90 cm tall. In India,* PL* is also known as Nagadaminee. It is a widespread species in the tropics and subtropics of the Old World. In Asia, it occurs from Arabia to India, Malaya, Indonesia, the Philippines, and New Guinea. It is also found in Egypt and throughout tropical Africa to S. Africa and Madagascar, in Australia, and elsewhere as an introduced weed. In Pakistan,* PL* occurs as a weed of dry sandy or stony waste ground, as a weed of cultivation, or on broken rocky hill slopes, ascending to at least 1000 m. It is an erect or straggling undershrub found in the hedges of fields, fruit orchards, dry scrub forests, and waste places of Kashmir to Kauman at an altitude of 300–1050 m and in all states of India [2].

In this study, the* PL* is selected due to ethnopharmacological information that the plant has been used to treat jaundice, abdominal colic, cephalgias, diarrhea, paralysis, erectile dysfunction, and vomiting [3]. The traditional medicinal importance of the plant is ascertained as the leaf paste of* PL* is an effective and inexpensive treatment of bone fracture for human beings as well as cattle.* PL* is useful in toothache and the stem is used as toothbrush traditionally in India [4]. Phytochemical analysis of* PL* foliage included 8 compounds, namely, 1- docosanol, stearic acid, stigmasterol, *β*-sitosterol, saropeptate (N-benzoyl-l-phenylalaninol acetate), *β*-sitosterol-3-0-d-glucopyranoside, stigmasterol-3-0-*β*-d-glucopyranoside, and 20-hydroxyecdysone [5]. Several pharmacological activities of plant reported by researchers are antioxidant [5], antinociceptive and antipyretic, antiplasmodial [3], and anticancer activity [6]; it also shows a beneficial effect in dog bite and rat bite [7]. Foliage parts of* PL* are used in form of poultices for healing of cuts and chronic wounds. A decoction of the black powder of the plant is drunk to cure piles and for enema, fever, and malaria. It is the first time that* PL* is evaluated for antidiabetic activity.

## 2. Materials and Methods

### 2.1. Collection, Authentication, and Extraction of Plant

The leaves of* PL* were collected in the month of March 2014 from the forest of Kanker, Chhattisgarh, India, authenticated by Dr. N. K. Satti, Department of Natural Products, Indian Institute of Integrative Medicine, Jammu, and leaves were deposited in the herbarium of the Institute. The powdered air-dried leaves (1 kg) of* PL* were extracted with ethanol (b.p. 40–60) by hot percolation. The extract was concentrated to dryness in a rotary evaporator (Buchi type) under reduced pressure and controlled temperature (37–40°C) and yield was found to be 16.2%. The dried extract was used to evaluate antiadipogenic, antidiabetic, and hypolipidemic activity.

### 2.2. Cell Lines

#### 2.2.1. Source of Cell Lines

3T3-Ll cell lines for evaluating antiadipogenic activity were received from National Centre for Cell Science (NCCS), Pune, India.

#### 2.2.2. Cell Culture and Maintenance for Adherent Cell Lines

Cells were grown in tissue culture flasks with complete growth medium at 37°C in an atmosphere of 5% CO_2_ and 90% RH in a carbon dioxide incubator. Cells were daily checked for their proper growth. The medium of the cells was changed when the color became yellow. To change the medium, the medium in the flask was aspirated with Pipette-Aid and discarded. The fresh medium (15–20 mL) was placed in the culture flask under sterile conditions. The flask was properly marked and incubated in CO_2_ incubator. Depending on the mass doubling time of cells, subculturing of cells was done, when they were at subconfluent stage.

#### 2.2.3. Subculture of the Cell Lines

It involves detachment of the cells from the growth surface (substratum) of the culture flask and inoculation of the cells into fresh medium in new culture flask, that is, TCF-25, TCF-75, or TCF-150 (depending on the number of cells). The medium of the flask at subconfluent growth was changed one day in advance. The entire medium from the flask was taken out and discarded. Cells were washed with phosphate buffer saline (PBS) (1 mL for TCF-25; 3 mL for TCF-75). Then Trypsin-EDTA (prewarmed at 37°C) was added (1 mL for TCF-25; 3 mL for TCF-75) and incubated for approximately 2 minutes at 37°C. Cell suspension was made with complete growth medium. An aliquot was taken out; cells were counted and checked for viability with Trypan blue. Cell stock with more than 98% cell viability was accepted for determination of* in vitro* cytotoxicity. The cell density was adjusted to 1 × 10^6^ cells mL^−1^ by the addition of more complete growth medium and inoculated into fresh TCF-75 or TCF-150 and incubated in CO_2_ incubator to continue the culture.

#### 2.2.4. Stages of Cell Growth during Culturing

**Figure 1:**
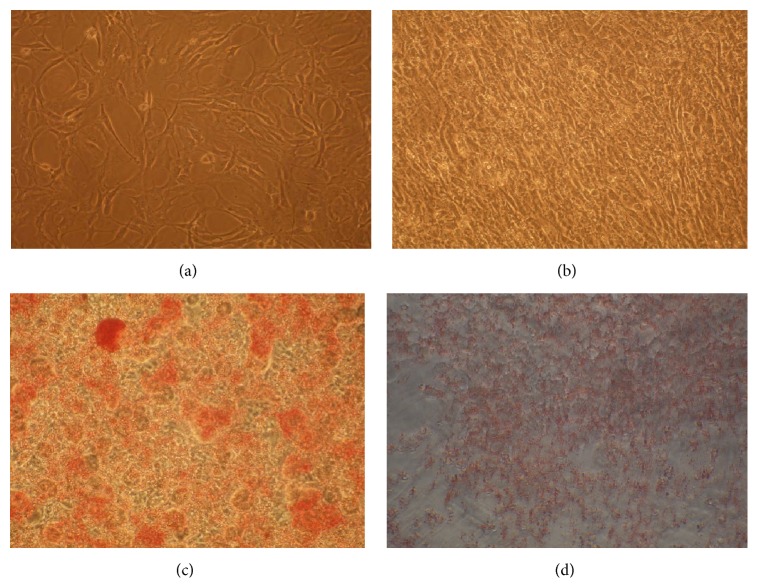
Different stages of cell growth. Figure 1 illustrates various growth stages of 3T3-L1 cell lines. (a) is the subconfluent cell stage; it is the nonadherent cell stage. (b) is confluent cell stage; it illustrates the adhesion of all cells in a culture disk. (c) is differentiated cell stage; it occurred when cells convert to specific cells. (d) is the inhibited cell stage; it comes when* PL* extract was poured on 3T3-L1 adipocytes during differentiated cell stage.

On observation under microscope, cells show roughly round shape when seeded in the culture flask. Different stages of cell growth during culturing are subconfluent, confluent, differentiated, and inhibited as shown in Figure 1.

### 2.3. Animals Used

The animals used in experiment were procured from animal house of Indian Institute of Integrative Medicine (IIIM) (CSIR), Jammu. Male Wistar rats weighing 160–200 g were taken. Experimental protocols were approved by the Institutional Animal Ethic Committee (IAEC reg number 65/99/CPCSEA/REG). The animals were kept in polycarbonate cages and maintained under standard housing conditions of temperature (22 ± 2°C) and humidity (45–64%) with 12 h light-dark cycle. Animals were fed pellet diet, purchased from Ashirwad Industries, Mohali, India, with supply of water* ad libitum* and normal saline. Animals were divided into five different groups as normoglycemic control, diabetic control, reference group, and test groups, AS001 (250 mg/kg) and AS002 (500 mg/kg) each containing six animals for* in vivo* antidiabetic and hypolipidemic activity.

### 2.4. Chemicals Used

The used chemicals were cholesterol (Qualigens Fine Chem., GSK, India), ethylenediamine tetra-acetic acid (EDTA; HiMedia Laboratories Pvt. Ltd., Mumbai), formaldehyde (Qualigens Fine Chem., GSK, India), isopropyl alcohol (Sisco Research Laboratories. Pvt. Ltd., Mumbai, India), and sodium bicarbonate (NaHCO_3_; HiMedia Laboratories Pvt. Ltd., Mumbai, India). The following chemicals were purchased from Sigma-Aldrich Inc., St. Louis, MO: dexamethasone, dimethyl sulfoxide (DMSO), Dulbecco's Modified Eagle Medium (DMEM), fetal bovine serum (FBS), 60 mg/L amikacin, gentamycin, 3-isobutyl-1-methylxanthine (IBMX), insulin, penicillin, phosphate buffer saline (PBS), Roswell Park Memorial Institute (RPMI) medium, streptomycin, Trypsin, Oil Red O dye, and sodium pyruvate. Assay kits for blood glucose, triglyceride, and total cholesterol were purchased from Siemens Medical Solutions Diagnostics Ltd., Baroda, Gujarat, India. Rat Insulin ELISA kit was purchased from Mercodia, Sweden. Reference drug like glibenclamide was generously provided by Nicholas Piramal Research Limited, Mumbai. Simvastatin and fenofibrate were purchased from local market. Streptozotocin and diabetogenic chemicals were purchased from Sigma Chemical Co., Germany.

### 2.5. Pharmacological Screening Methods

#### 2.5.1. *In Vitro* Experimental Method

*In Vitro Cytotoxicity Assay*. 3T3-L1 cell line was cultured in DMEM with 10% fetal bovine serum (FBS) and 60 mg/L amikacin at 37°C, 5% CO_2_, and above 90% relative humidity. The* in vitro* toxicity of ethanolic extract in a pane of cell line 3T3-L1 was assayed by Neutral Red assay. The cells were cultured in 150 cm^2^ culture flasks in medium supplemented with FBS (10%) and amikacin (60 mg/L) at 37°C, 95% humidity, and 5% CO_2_ and subcultured every 2-3 days using standard cell culture techniques. For Neutral Red assay, cells were seeded as 2.5 × 10^4^ cells/well and allowed to grow undisturbed for 24 h at 37°C. Cell counts were made by using the Trypan blue exclusion method to determine cell viability. Test extracts were diluted appropriately in DMEM complete and incubated for 48 h. The number of viable cells was determined using Neutral Red assay procedure. Cell containing sample extract was exposed to extract. After incubating with samples, the cells were washed with saline and incubated for 90 min with the medium containing Neutral Red (166 *μ*g/mL). The cells were washed to remove extracellular dye. A solution of acidified isopropanol (0.33% HCl) was then added to lyses the cells; as a result, the incorporated dye was liberated from the viable cells. The absorbance was measured at 540 nm on a Microplate Reader (Molecular Devices, SPECTRAMAX plus 384). All the assays were performed in triplicate.

**Figure 2:**
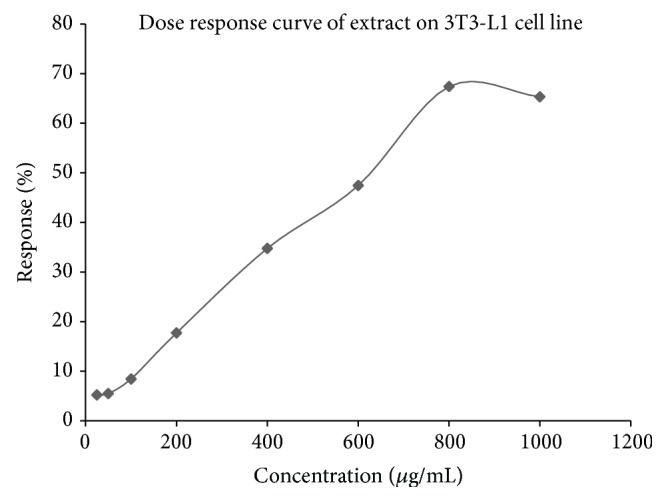
Dose response curve of extract for 3T3-L1 cell line. Values are expressed as mean ± SEM (number of experiments, *n* = 6). Dose response curve of the extract revealed poor discrimination at 25 *μ*g/mL and showed greater sensitivity at 200 *μ*g/mL. Ceiling effect observed at 1000 *μ*g/mL where no more increase in the response is seen with further increase in the dose. Extract exhibited the inhibitory effect with an inhibitory concentration (IC_50_) of 662.14 *μ*g/mL.

*Dose Response Curve for the Toxicity Studies*. Dose response curve of the extract for 3T3-L1 cell line is shown in Figure 2. The inhibitory concentration (IC_50_) of the extract was found to be 662.14 *μ*g/mL.

*3T3-L1 Adipocyte Differentiation*

**Step 1 (subculture and seeding of preadipocyte cell line for induction of differentiation).**

When the cells are around 70% to 80% confluent, cells were harvested from 25 mm tissue culture flask by trypsinization. Seed the cells in complete media on desired tissue culture vessel like 96/24/12/6-well plates. The cells were allowed to grow to 100% confluency (2 days). Cells were kept for another 48 hours in this state to arrest the cell division; then cells were treated with adipocyte differentiation media.

**Step 2 (adipocyte differentiation).**

After postconfluency, adipogenesis (adipocyte differentiation) was performed by adding differentiation medium which consists of insulin (1 mg/L), isobutyl-1-methylxanthine (0.5 mM/L), and dexamethasone (0.25 *μ*M/L). Test extracts concentration (50, 100, and 200 *μ*g/mL) at day 0 and keep the cells for 96 hours in this state.

**Step 3 (adipocyte maturation).**

After postconfluency, discard differentiation medium by gentle pipetting. In this stage, cells can be easily detached from plate. Add adipocyte maturation media containing insulin (1 mg/L) in control group and* PL* extract (50, 100, and 200 *μ*g/mL) in treated group. Maturation media were changed every 2 days followed by addition of fresh media. After day 8, lipid droplets inside the cells were visible and around day 14 large lipid droplets were found. After day 8, cells were used for assay. Cells can be kept for around 1 month in this condition by changing media every 2 days.

**Step 4 (adipocyte differentiation conformations by Oil Red O staining and optical density (OD) measurement).**

Oil Red O dye was eluted by adding 100% isopropanol and then incubated for 10 min. Pipette the isopropanol with Oil Red O up and down several times to be sure that all Oil Red O is in the solution. Transfer 1 mL solution to the cuvette and measure OD at 520 nm in spectrophotometer; as blank, use 100% isopropanol. OD of nondifferentiated cells is 0.05 and OD of differentiated cells is 0.7 to 1.0.

#### 2.5.2. *In Vivo* Experimental Methods

*Acute Oral Toxicity Study*. Wistar male rats weighing 150–200 g were used for acute toxicity study. The toxicity study was performed by administering extract orally in different doses of 150, 500, 1000, and 2000 mg/kg as per OECD guideline 423. All rats were observed for pertinent behavioral changes and signs of toxicity daily that included changes in skin, fur, eyes, and mucous membranes, occurrence of secretions and excretions, autonomic activity (e.g., lacrimation, piloerection, pupil size, and unusual respiratory pattern), and changes in gait, posture, response to handling, as well as the presence of clonic or tonic movements. Mortality was not found up to 2000 mg/kg dose, so half of the maximum dose was considered for therapeutic exploration.

*18 h Fasted Rat Model*. Male Wistar rats in each group were fasted overnight. The animals were divided into normoglycemic control, test groups, and reference group. Normal animals received Tween 80 solution (1 mL/kg), test groups received test extract (250 and 500 mg/kg), and reference group received glibenclamide (5 mg/kg). Blood glucose determination was done at 0 h (prior to any treatment) and 3 h (after drug administration) on the same day [8].

*Oral Glucose Tolerance Test (OGTT)*. Male Wistar rats in each group were fasted overnight. The animals were divided into normoglycemic control, negative control (glucose primed), test treated (glucose primed + extract), and reference treated (glucose primed + glibenclamide) group. Normal and negative control groups received Tween 80 solution (1 mL/kg), test groups received* PL* extract (250 and 500 mg/kg), and reference group received glibenclamide (5.0 mg/kg). The test and reference drug was administered at 0 h before glucose treatment. Glucose (1.5 g/kg, 10% solution) was administered to all groups except normoglycemic control group. Blood glucose level was determined at 0 min, 30 min, and 90 min after glucose administration [9].

### 2.6. STZ Induced Diabetic Rat Model

#### 2.6.1. Induction of Diabetes in Rats

Diabetes mellitus was induced by single intraperitoneal (i/p) injection of freshly prepared STZ (45 mg/kg) in 0.1 M citrate buffer (pH 4.5) in a volume of 1 mL/kg body weight. Diabetes was established in STZ treated rats over a period of 7 days. After 7 days, the blood was collected by retroorbital route and the plasma glucose level of each rat was determined. Animals with fasting blood glucose (FSG) of range 250–300 mg/dL were considered diabetic and included in the study. Blood sample was collected on 0, 7, 14, 21, and 28 days. Normal chaw fed and normal saline were given daily [10].

#### 2.6.2. Treatment Protocol for STZ Induced Diabetic Rat Model

Experimental animals were divided into five groups each containing six animals. Normal group received 1% v/v Tween 80 (1 mL/kg); diabetic control group (STZ, 45 mg/kg), reference group (glibenclamide, 5.0 mg/kg), and test groups received extract of* PL* at two different doses of 250 mg/kg (AS001) and 500 mg/kg (AS002) in 1% v/v of Tween 80 (1 mL/kg). The treatment protocol was continuing for period of 28 days.

### 2.7. Bioassays

#### 2.7.1. Blood Glucose

Blood glucose level was estimated by glucose oxidase/peroxidase (GOD/POD) method using commercially available enzymatic kit from Bayer Diagnostics India Ltd., Baroda, Gujarat, India [11].

#### 2.7.2. Triglyceride (TG)

Triglycerides are the chemical form in which most fat exists in food as well as in the body. TG was determined by enzymatic colorimetric method [12, 13].

#### 2.7.3. Lipid Profile

Lipid profiles including total cholesterol (TC), high density lipoprotein (HDL), and low density lipoprotein (LDL) were estimated by the enzymatic method as described by Allain et al., 1974 [14].

#### 2.7.4. Insulin ELISA

Insulin ELISA has been done as per the instruction of manufacturer (Mercodia, Sweden) [15–17].

### 2.8. Histopathological Study

Pancreatic tissues were isolated from all group animals and subjected to histopathological study postexperimentally. The whole pancreas from each animal was removed after sacrificing the animal under chloral hydrate (400 mg/kg, i/p) anesthesia. Pancreas was collected in formaldehyde solution and immediately histological preparations were made and 0.5 *μ*g thick sections were cut and stained with haematoxylin and eosin for histological examination [18].

### 2.9. Statistical Analysis

All values were expressed as mean ± SEM. The results were analyzed statistically using one-way analysis of one-way variance (ANOVA) followed by Dennett's *t*-test to calculate the level of significance. Values are expressed as mean ± SEM (number of animals, *n* = 6), significantly different at ^*∗∗*^ *p* < 0.05, ^*∗∗∗*^ *p* < 0.001, when compared with diabetic control group.

## 3. Results

### 3.1. *In Vitro* Toxicity of Ethanolic Extract of* PL* on 3T3-L1 Cell Lines

*In vitro* toxicity of* PL* extract was performed on 3T3-L1 cell lines by Neutral Red assay. The extract was taken at the concentration of 25, 50, 100, and 200 *μ*g/mL. It was observed that more than 75% of cells were viable on maximum dose, thus indicating the safe nature of the extract. The inhibitory concentration (IC_50_) of the extract was found to be 662.14 *μ*g/mL. The dose response curve for* PL* extract is shown in Figure 2.

### 3.2. Effect of* PL* Extract on* In Vitro* Adiposity Differentiation Assay

The extracts of* PL* showed different results on different concentration in adipocyte differentiation assay. The results showed more than 50% inhibition in differentiated cell as compared to nondifferentiated cell. The percentage of inhibition caused by* PL* extract is shown in Table 1.

### 3.3. Acute Toxicity Study of* PL* Extract

Acute toxicity study revealed that* PL* extract did not produce any toxic symptoms and mortality when administered to rats at a dose up to 2000 mg/kg. There was no sign of change in skin, fur, eyes, and mucous membrane; along with that, autonomic activity, gait, and posture were also unaffected. The study till the dose level of 2000 mg/kg is safe. As per acute toxicity, result doses of 250 and 500 mg/kg were used for* in vivo* antidiabetic study.

### 3.4. Evaluation of Antidiabetic Activity

#### 3.4.1. Hypoglycemic Effect of Ethanolic Extract of* PL* in 18 hr Fasted Rat Model

**Figure 3:**
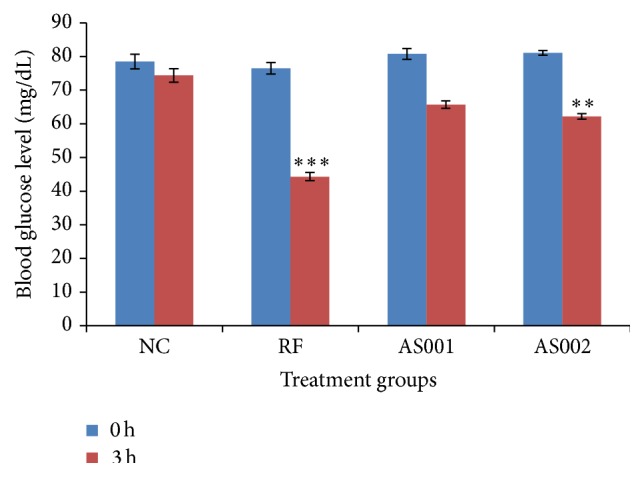
Effect of* Pupalia lappacea* extract on 18 h fasted rats. Values are expressed as mean ± SEM (number of animals, *n* = 6); NC = normoglycemic control, RF = reference group (glibenclamide, 5.0 mg/kg), significantly different at ^*∗∗*^ *p* < 0.05, ^*∗∗∗*^ *p* < 0.001 when compared with diabetic control group.

A preliminary study was performed to determine hypoglycemic activity of the extract in 18 h fasted rat model. The test groups showed significant fall in fasting serum glucose (FSG) level, that is, 65.7 and 62.2 mg/dL, for AS001 and AS002, respectively. Blood glucose level with hypoglycemic effect of* PL* extract is showed in Figure 3.

#### 3.4.2. Oral Glucose Tolerance Test (OGTT)

Oral glucose tolerance test was performed in overnight fasted rats to observe the potency of extract in glucose primed condition. Both the test groups exhibited fall in FSG level, that is, 119 and 110 mg/dL, at 90 min for AS001 and AS002, respectively, in glucose primed condition. However, reduction in blood glucose level after glibenclamide treatment was found to be 96 mg/dL. This reduction in FSG level was more for reference group as compared to test groups. The effect of* PL* extract on blood glucose level is shown in Table 2.

#### 3.4.3. STZ Induced Diabetes Model

*Effect of PL on Fasting Blood Glucose Level*. Administration of* PL* extract in STZ induced diabetic rats caused significant reduction in FSG level in both the test groups. Fall in blood glucose level by test group AS002 was found to be 211.61 mg/dL which was higher than the other test group AS001. This reduction in FSG level by AS002 was comparable with the reference drug, glibenclamide. The effect of* PL* extract on fasting blood glucose level in STZ induced diabetic rats is shown in Table 3.

*Effect of PL on Insulin Level in Diabetic Rats*. Administration of extract in diabetic animals caused significant increase in the level of insulin at day 28. The rise in the level of insulin was significant with all treated groups, that is, reference group (0.58 mg/dL) and test groups AS001 (0.50 mg/dL) and AS002 (0.57 mg/dL). The level of insulin at day 1 was the initial reading; however insulin level at day 28 was final reading.  Table 4 summarizes the effect of* PL* extract on insulin level in diabetic rats.

**Figure 4:**
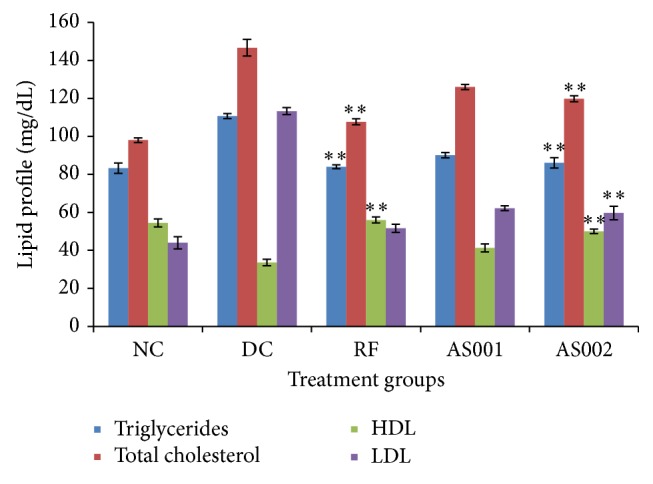
Effect of* Pupalia lappacea* extract on lipid profile of STZ induced diabetes in rats. Values are expressed as mean ± SEM (number of animals, *n* = 6); NC = normoglycemic control, DC = diabetic control, and RF = reference group (glibenclamide, 5.0 mg/kg), significantly different at ^*∗∗*^ *p* < 0.05, ^*∗∗∗*^ *p* < 0.001 when compared with diabetic control group.

*Effect of PL Extract on Lipid Profile of STZ Induced Diabetes in Rats*. The improvement in lipid profile by* PL* extract also supports its antidiabetic activity along with antihyperlipidemic activity. Improvement in lipid profile reduces the possibilities of diabetes induced obesity and cardiovascular diseases. Administration of extract in diabetic animals caused significant increase in the level of high density lipoprotein (HDL) and restores the level of total cholesterol (TC), triglyceride (TG), and low density lipoprotein (LDL) near to the normal value. However, increase in the HDL level in reference group was more than test groups.  Figure 4 suggests the effect of* PL* extract on lipid profile of diabetic rats.

*Effect of PL Extract on Body Weight of STZ Induced Diabetes in Rats*. Daily administration of extract for 28 days to STZ induced diabetic rats caused a significant rise in body weight which was comparable with the reference drug. The effect of* PL* extract on body weight of diabetic animals is shown in Table 5.

### 3.5. Histopathology

Results showed that the extract enhanced the regeneration of islets of Langerhans in the pancreas and restoration of normal cellular size of the islet with hyperplasia. In this study, the damage of pancreas in STZ treated diabetic control rat and regeneration of islets of Langerhans by glibenclamide were observed. The comparable regeneration and restoration of normal cellular size of the islet with hyperplasia were also shown by ethanolic extract of* PL*. The hypoglycemic effect may be attributed to the regeneration of islet of Langerhans which may confirm the efficiency of the given extract in the management of diabetes.

## 4. Discussion

The research protocol was envisaged and designed to evaluate the antidiabetic and antiadipogenic efficacy of* PL* with a main focus on the controlling of hyperglycemic and dyslipidemic conditions. Literature survey conducted on this plant revealed no antidiabetic property, since, in the diabetes management, different aspects in the glucose metabolism play vital role. For instance, the rate of glucose absorption, insulin secretion in response to high glucose in the blood, and the insulin utilization/action under insulin resistance state were studied. Thus, with the advancement of research in the area, a wide range of parameters and experimental targets have been studied to assess the overall potential of test material against diabetes. The protocol was designed in such a way to cover these parameters broadly. Two mechanistically different research techniques were employed for the evaluation of biological effect of the test extract on insulin secretion in Wistar rats. The first was* in vitro* adipocyte differentiation assay on 3T3-L1 for evaluating antiadipogenic activity and the second was* in vivo* experimentation including 18 h fasted normal rats, glucose primed rats, and STZ induced diabetic rats employed to explore if this test plant extract possesses any antidiabetic potential.

Before screening any compound for biological activity, we need to test the toxicity of the compound. Hence* in vitro* and* in vivo* toxicity studies were conducted to determine the suitable dose for administration. For* in vitro* study, the extract was found to be safe due to its nontoxic nature at highest dose (200 *μ*g/mL) on 3T3-L1 cell lines. However for* in vivo* study the* PL* ethanolic extract up to 2000 mg/kg dose did not reveal any physical signs of toxicity or mortality even after 2 weeks of treatment; it can be considered relatively safe and hence two doses of the extract were used for detailed* in vivo* study. To determine antiadipogenic activity of test extract, an* in vitro* adiposity differentiation assay was conducted using 3T3-L1 cell lines. In normal condition 3T3-L1 preadipocyte cells have fibroblastic phenotype. When this cell was treated with differentiation media, they accumulate lipid droplet inside the cell and achieve adipocyte phenotype. The* PL* extract caused significant inhibition of adipocyte cells and inhibition was more in differentiated cells as compared to nondifferentiated cells. This finding confirmed the antiadipogenic effect of* PL* extract. While determining* in vivo* antidiabetic activity using 18 h fasted rat model, the* PL* extract also exhibited significant results. Both the test groups, that is, AS001 (250 mg/kg) and AS002 (500 mg/kg), showed dose dependent fall in fasting serum glucose (FSG) level. In addition to this animal model, the oral glucose tolerance test (OGTT) model also showed dose dependent fall in blood glucose level by* PL* extract in glucose primed condition rats. In this experimental model, the fall in serum glucose level after 90 min was more in reference group than test groups. However,* PL* extract also exhibited fall in serum glucose which was near to reference group. This dose dependent fall in FSG suggested that* PL* extract possesses definite hypoglycemic property.

Moreover, the daily administration of* PL* ethanolic extract to STZ induced diabetic rats for 4 weeks caused a significant reduction in serum glucose level and an increase in the lost body weight. STZ, a highly cytotoxic agent of pancreatic *β* cells [19], induces diabetes by damaging the cells which causes reduction in insulin release. It is reported that treatment of diabetic animals with medicinal plant extracts resulted in activation of *β* cells and granulation returned to normal, showing an insulinogenic effect [20]. The possible mechanism through which PL ethanolic extract possessed antihyperglycemic effect might be due to increased release of insulin from remnant *β* cells and/or regenerated *β* cells. In this context, a number of other plants have been reported to have antihyperglycemic activity with a stimulatory effect on insulin release [20]. Since the extract produced significant antihyperglycemic effect in rats in which most of the *β* cells are damaged, it is likely that extract might have extra pancreatic mechanism of action. The elevation of serum insulin in extract treated diabetic rats could be due to either the insulinotropic substances present in the extract, which induce the intact functional *β* cells to produce insulin, or the protection of the functional *β* cells from further deterioration so that they remain active and produce insulin. Similarly the extracts of* Medicago sativa* [21],* Eucalyptus globulus* [22], and* Sambucus nigra* [23] have been shown to possess insulin-releasing action both* in vitro* and* in vivo*. Since insulin inhibits the activity of Glc-6-Pase in the liver of STZ induced diabetic rats and controls hepatic glucose production (HGP), the insulinotropic effect of* PL* extract might play a crucial role in the control of hyperglycemia in STZ diabetic rats. The suppression of Glc-6-P hydrolysis could also be one of the reasons for the hypoglycemic effect of* PL* extract in STZ induced diabetic rats.

Diabetic animals treated with extract exhibited significant rise in the body weight. The ability of extract to recover body weight loss seems to be due to its antihyperglycemic effect. In diabetes, hyperglycemia is accompanied with dyslipidemia that is characterized by increase in TC and TG. This altered serum lipid profile was reversed towards normal after treatment with the extract.* PL* extract exhibited hypocholesterolemic and hypotriglyceridemic effects, while it increased the levels of HDL in diabetic rats. However, extract was found to be more effective in reducing the levels of TG and LDL as compared to its effect on TC and HDL.

## 5. Conclusion

The* PL* extract was screened at different dosages for the* in vitro* and* in vivo* experiments. With a focus on the therapeutic requirement of the antidiabetic treatment, the experimental targets and parameters were decided. The main findings of the research work on the test plant extract were that 500 mg/kg dose of ethanolic extract of* PL* prevented a rise in blood glucose level in glucose primed rats and STZ treated diabetic animal and also showed maximum inhibition in adipogenic activity. Furthermore, 500 mg/kg dose of extract of* PL* showed significant antidyslipidemic effect in STZ induced rats. It improved the lipid profile by decreasing the levels of serum TG, TC, and LDL and increasing HDL as compared to reference drug. Based on the observations made from these studies, it is concluded that not only the blood glucose but the lipids profile of the organism bears an equal relevance to diabetes because diabetes appears to involve dyslipidemia. The relevance of antiobesity mechanism, in this context, may be more appropriate. It is concluded from the data that the extract possesses potent antihyperglycemic and antiadipogenic activity and it may prove to be effective for the treatment of diabetes. Further studies to isolate, identify, and characterize the active principle(s) of the extract are in progress.* PL* has a lot of future scope in regard of further research work carried out for the treatment of endocrine disorder like diabetes by herbal drugs supplements without involvement of any side effects.

## Figures and Tables

**Table 1:** Antiadipogenic activity of different dose of extract in 3T3-L1 cell line.

S. number	Treatment group	% of inhibition		
		Concentration (*µ*g/mL)		
		50	100	200
1	AS001	18.81 ± 0.60	22.84 ± 0.69	23.51 ± 0.30^*∗∗*^
2	AS002	15.34 ± 0.26	24.01 ± 0.31	29.18 ± 0.70^*∗∗∗*^
3	Simvastatin	44.84 ± 1.21	60.84 ± 0.69	64.18 ± 0.86

Values are expressed as mean ± SEM (*n* = 6), significantly different at ^*∗∗*^ *p* < 0.05,  ^*∗∗∗*^ *p* < 0.001.

**Table 2:** Effect of *Pupalia lappacea* in blood glucose primed rats.

S. number	Treatment group	Dose (mg/kg)	Blood glucose level (mg/dL)		
			0 h	30 min^#^	90 min^#^
1	NC	Untreated	78 ± 3.7	76 ± 2.9	74 ± 1.9
2	GP	Vehicle	87 ± 3.3	184 ± 6.3	152 ± 4.3
3	RF	5.0	90 ± 3.9	152 ± 3.2^*∗∗∗*^	96 ± 3.8^*∗∗∗*^
4	AS001	250	86 ± 2.9	153 ± 6.2^*∗∗*^	119 ± 2.6^*∗∗*^
5	AS002	500	88 ± 2.8	151 ± 3.2^*∗∗∗*^	110 ± 1.3^*∗∗∗*^

NC = normoglycemic control, GP = glucose primed, and RF = reference group (glibenclamide, 5.0 mg/kg). Values are expressed as mean ± SEM (number of animals, *n* = 6), significantly different at ^*∗∗*^ *p* < 0.05, ^*∗∗∗*^ *p* < 0.001 when compared with diabetic control group, # refers to “Time post glucose administration”.

**Table 3:** Effect of *Pupalia lappacea* in blood glucose level of STZ induced diabetes in rats.

S. number	Treatment group	Dose (mg/kg)	Blood glucose level (mg/dL)				
			Day 0	Day 7	Day 14	Day 21	Day 28
1	NC	Untreated	78.67 ± 2.51	92.61 ± 5.01	90.78 ± 12.49	88.46 ± 4.38	84.26 ± 5.48
2	DC	Vehicle	80.03 ± 3.60	364.27 ± 16.17	351.62 ± 21.92	338.12 ± 17.24	340.68 ± 11.24
4	RF	5.0	76.83 ± 8.52	344.21 ± 9.58	340.01 ± 14.65	261.36 ± 3.11^*∗∗*^	119.59 ± 9.75^*∗∗*^
5	AS001	250	84.04 ± 3.14	361.83 ± 6.37	341.60 ± 18.86	303.21 ± 6.58	234.68 ± 5.74
6	AS002	500	78.78 ± 3.88	346.53 ± 2.30	333.11 ± 17.12	284.82 ± 6.05	211.61 ± 3.41^*∗∗∗*^

NC = normoglycemic control, DC = diabetic control, and RF = reference group (glibenclamide, 5.0 mg/kg). Values are expressed as mean ± SEM (number of animals, *n* = 6), significantly different at ^*∗∗*^ *p* < 0.05, ^*∗∗∗*^ *p* < 0.001 when compared with diabetic control group.

**Table 4:** Effect of *Pupalia lappacea* on insulin level of STZ induced diabetes in rats.

S. number	Treatment group	Insulin level (mg/dL)	
		Initial reading	Final reading
1	NC	0.86 ± 0.02	0.87 ± 0.48
2	DC	0.89 ± 0.01	0.26 ± 0.04
3	RF	0.88 ± 0.021	0.58 ± 0.17
4	AS001	0.89 ± 0.031	0.50 ± 0.57
5	AS002	0.85 ± 0.034	0.57 ± 0.24^*∗∗*^

NC = normoglycemic control, DC = diabetic control, and RF = reference group (glibenclamide, 5.0 mg/kg). Values are expressed as mean ± SEM (number of animals, *n* = 6), significantly different at ^*∗∗*^ *p* < 0.05 when compared with diabetic control group.

**Table 5:** Effect of *Pupalia lappacea *in body weight of STZ induced diabetes in rats.

S. number	Treatment group	Dose (mg/kg)	Body weight (g/kg)				
			Day 0	Day 7	Day 14	Day 21	Day 28
1	NC	Untreated	185 ± 1.3	190 ± 1.5	196 ± 2.4	201 ± 2.1	209 ± 1.3
2	DC	Vehicle	188 ± 2.5	182 ± 2.7	164 ± 2.8	123 ± 3.1^*∗∗*^	107 ± 2.5
4	RF	5.0	188 ± 1.2	179 ± 2.3	172 ± 2.6	176 ± 2.3^*∗∗∗*^	186 ± 2.1^*∗∗∗*^
5	AS001	250	186 ± 3.1	185 ± 3.1	168 ± 3.3	167 ± 2.7	168 ± 3.6^*∗∗*^
6	AS002	500	187 ± 6.1	174 ± 2.9	169 ± 3.2	175 ± 1.9^*∗∗∗*^	183 ± 2.6^*∗∗∗*^

NC = normoglycemic control, DC = diabetic control, and RF = reference group (glibenclamide, 5.0 mg/kg). Values are expressed as mean ± SEM (number of animals, *n* = 6), significantly different at ^*∗∗*^ *p* < 0.05, ^*∗∗∗*^ *p* < 0.001 when compared with diabetic control group.
